# The ethics of everyday practice in primary medical care: responding to social health inequities

**DOI:** 10.1186/1747-5341-5-6

**Published:** 2010-05-03

**Authors:** John S Furler, Victoria J Palmer

**Affiliations:** 1 Primary Care Research Unit, The Department of General Practice, The University of Melbourne, 200 Berkeley St, Carlton, VIC Australia 3053

## Abstract

**Background:**

Social and structural inequities shape health and illness; they are an everyday presence within the doctor-patient encounter yet, there is limited ethical guidance on what individual physicians should do. This paper draws on a study that explored how doctors and their professional associations ought to respond to the issue of social health inequities.

**Results:**

Some see doctors as bound by a notion of care that is blind to a patient's social position, while others respond to this issue through invoking notions of justice and human rights where access to care is a prime focus. Both care and justice orientations however conceal important tensions linked to the presence of bioethical principles underpinning these. Other normative ethical theories like deontology, virtue ethics and utilitarianism do not provide adequate guidance on the problem of social health inequities either.

**Conclusion:**

This paper explores if Bauman's notion of "forms of togetherness" provides the basis of a relational ethical theory that can help to develop a response to social health inequities of relevance to individual physicians. This theory goes beyond silence on the influence of social position of health and avoids amoral regulatory approaches to monitoring equity of care provision.

## Background

The socially patterned nature of disease and illness is now a commonplace understanding of health [1]. Social disadvantage and vulnerability, differences in income, occupational group and status, quality of housing and level of education are not just distal and remote influences on the health of a community. The impact of such influences is felt by individuals, real patients who present in clinical practice. Each of us takes our place in the inequitable social gradient of health, embodying a lifetime of socially patterned resources, choices and relationships that create health and illness. The unequal lives of patients and the health effects of social disadvantage are thus a daily reality of medical care. The family physician particularly cannot avoid the effects of social disadvantage in their ongoing relationship with their patients. How to respond to these inequities is challenging and there is a lack of adequate ethical guidance on the matter.

Primary care occupies a unique place in the effort to understand how physicians might respond to this issue as it is so closely linked to the social world of the patient. The primary care physician provides diagnosis, referral, coordinates patient care, interprets health care systems and translates complex information for patients in their everyday community setting. Ideally the relationship between patient and primary care physician is ongoing and longitudinal, where doctors know their patients from cradle to grave, a relationship premised on deep biographical knowledge of the person in the context of family and community [2,3]. Increasingly fragmented and discontinuous care [4] means that this idealised relationship is under pressure, both in countries where primary care has traditionally played a first contact "gate keeping" role as well countries such as the United States where this is not always the case. Nevertheless, across health systems primary medical care remains community-based and generalist in focus.

Encounters with patients in primary care are thus an unfiltered, complex meld of biology, psychology, historical and contemporary social and societal influences. Family physicians negotiate this complexity and uncertainty as they account for the social, cultural and community context of their patients in their diagnosis. It is in these day to day encounters between family physician and patient where ineluctably, the lives of individuals intersect with the macro social and structural injustices that permeate health experiences [5]. The primary care medical encounter offers a particularly useful place to begin an exploration of how physicians ought to respond to the moral problem of social health inequities. A response seems warranted given that these issues are present in everyday encounters between family physicians and patients. Moreover, the delivery of medical care is not only a scientific endeavour, but also a humanistic and moral pursuit [6].

The family doctor and patient relationship has received a great deal of attention in research. Ethics, sociology, anthropology, philosophy and other fields of enquiry have all struggled to understand this unique relational setting. The doctor-patient relationship is now the subject of intense interest within a "thriving multidisciplinary community of practitioners, educators and researchers" [[5]:434]. Primary care research has particularly led this enterprise [7]. Much of this interest and study has been driven by a growing perception that medical care has become technical, mechanistic, dehumanised and reductionist. The result has been an appreciation of the doctor-patient relationship as unique and one that takes on almost sacred qualities because the private suffering of individuals is laid bare, complete with the troubles of failing and deteriorating human bodies.

Yet the focus on the relationship between physician and patient has tended to push out of frame the important structural and social factors shaping the health experiences of patients. While many studies have examined physician-patient communication [8-10], how social health inequities are responded to or avoided within the clinical encounter has been overlooked. Social health inequities remain the "elephant in the room" [11], unacknowledged and silenced (intentionally and unintentionally) through collective endeavour. Four common responses from physicians to the issue of social inequities have been described which assist in silencing this issue [12]. Some blame the victim for their disadvantage (a common view across society as a whole). Some feel sympathy but do not see it as part of their job to address social problems. Some feel powerless to do anything about wider forces operating in a patient's life. Others address social disadvantage as best as they can in an adhoc way. While each response has a rationale, together they foster collective professional silence on its presence in the encounter. This means that clear guidance on how physicians might respond has not emerged.

In this paper we advocate for more active engagement with social health inequities as an everyday aspect of primary medical care. Our view is that commonly drawn upon normative ethical theories such as utilitarianism and deontology, and the four bioethical principles -- respect for justice, beneficence, non-maleficence and autonomy -- still keep social health inequities as an elephant in the room. This is reflected in the common responses to the problem outlined above. In this paper we argue that 'three forms of togetherness' described by sociologist Zygmunt Bauman can help to understand and develop a response to social health inequities. These forms of togetherness are described as *being-aside*, *being-with *and *being-for *[13]. The ideal relationship *being-for *builds on the ethical theory of philosopher Emmanuel Levinas [14] and illustrates a relational approach to ethics applicable to the medical encounter. It is our view that a relational approach to social health inequities can overcome some of the limitations of other normative ethical theories and the traditionally applied four bioethical principles.

## Methods

To examine this moral dilemma, we present a re-analysis of key findings of a study completed in 2002 that explored how Australian family physicians and their national professional association, the Royal Australian College of General Practitioners (RACGP), should or could respond to the issue of social inequities in health [15]. The study involved a review of policy and program documents relevant to the education, training, standard setting and accreditation programs of the College. 80 semi-structured interviews with key representatives from national committees, other family practice peak bodies, heads of academic family practice university departments, consumer groups and quality assurance organisations were conducted. Data was also collected from two focus groups held with 11 practicing primary care physicians from both rural and urban settings of Australia. All data collection focussed on the question of how the profession should respond to the issue of social health inequities.

Although the study had implications for both professional medical associations and individual physicians, this paper focuses on the role of individual physicians in their routine medical practice. While the term "social disadvantage" can encompass a wide range of cultural, contextual and socioeconomic factors, this study focused primarily on socioeconomic factors, and we retain that focus in this paper. In this re-analysis, the initial study findings are re-examined to identify the ethical limitations of participants' views about how the profession should respond to social health inequities. We combine this with a critique of other existing normative ethical theories such as virtue ethics, deontology and utilitarianism and their limitations for addressing social health inequities. We conclude with a theoretical exploration of Bauman's "three forms of togetherness" as a way to develop a response to this moral dilemma.

## Results: Two moral orientations and the survival of bioethical principles

The initial study results [15] found critical differences in the values that different groups expressed on social health inequities and physician responsibility. Participant responses revealed two opposing moral orientations closely related to those described by Gilligan of "care" and "justice". Gilligan's [16] term "moral orientation" describes how women reason about moral dilemmas using an ethic of care, while men's reasoning draws on justice and rights. Gilligan's argument is that women express a "different moral voice" to men. According to Friedman, [[17]:117] this voice has been overlooked in "the typical moral perspective...oriented toward matters of justice and rights and dominated by abstract principles". Self et al summarise the two moral orientations:

"[P]ersons who exemplify a justice orientation view relationships in terms of inequality versus equality and in terms of mutuality and reciprocity. One who adopts this orientation asks, "What is fair for all involved in a situation"? Justice thus connects vulnerability with oppression...By contrast, from the orientation of care, relationships are characterised not by equality and inequality but attachment versus detachment. Care connects vulnerability with the moral issues of support versus abandonment, not with oppression and inequality" [[18]:55].

In this study, participants expressed both orientations of justice and care, but they were not linked to gender.

Little [[19]:192], in line with Friedman, suggests the orientation of justice in particular appeals to impartial principles. Justice emphasises the importance of deliberating from a dispassionate stance where moral issues are dealt with by referring to rights of equality and non-interference. As an example, a family physician talking with a patient about the patient's injecting drug use could see this as primarily an issue of autonomy. Information should be given to the patient about drug related harm and potential impacts on health and clearly they should be advised to take precautions to avoid harm. However, in the end the issue of the patient's social world as an important factor can be silenced if this is seen simply as a matter of free-will and the patient's right to choose. Not interfering by deciding not to give information about the impacts and harms of drug use appears to be ethically justifiable because to intervene is to impinge on the individual's autonomy.

In contrast, the care orientation resists closure; it is sceptical about algorithmic principles, is receptive to details of context and values, and reasons through a stance of emotional engagement. Care connects a person with a moral responsibility to respond. In the patient's drug use example, the issue cannot be reduced to a question of rights and choices but rather considered as a contextual reality affecting and interacting with the individual's health. Because of the relationship between social disadvantage and adversity, injecting drug use and health outcomes there is the possibility and opening for patient and doctor to bring this complex problem into their encounter, which does not necessarily end if the patient continues drug use. In the care orientation the boundaries of responsibility flow out as the physician is seen as needing to respond to all aspects of patient health not just the disease and illness.

As Little [19] highlighted moral orientations do not automatically translate to moral theories for action. From an ethical perspective, Gilligan's care and justice orientations may be more "fruitful material for exploring ... the ethics of normatively substantive relationships", relationships characterised by "normative essence" or "normatively construed telos" [16]. Little argues that the orientations are best understood as "gestalts" that shape perceptions of self and other and are active in the way nuances, preferences and attitudes shape day-to-day behaviour.

The manner in which orientations shape perceptions of self and other is illustrated in participant responses in the study. For example, some participants contested the notion that the task of medicine lies beyond the physician's immediate, natural and essential responsibility to address individual suffering. It is the individual patient's and his/her illness that is responsible. Acting to address the social inequity and disadvantage embodied in a patient's presentation was outside of the profession and individual doctors' responsibility. This group of participants acknowledged that the College could (but had no responsibility to) help to raise awareness about the issue amongst family physician members. They suggested developing educational resources and learning activities focused on health inequity. Exposing new trainee family physicians to work in socio-economically disadvantaged settings was thought to be valuable. However, the moral responsibility of family physicians and the profession was one of simply providing each individual patient with high quality clinical care. To demonstrate:

"[M]y personal feeling is that the College should be about training general practitioners [family physicians] and keeping its members as expert as possible. I don't know that the College really needs to take on a role beyond that in term of society's wellbeing. It does that by providing good doctors" (Focus Group GP Participant).

Quite clearly, addressing distal notions of societal causes of illness and disease is secondary and incidental to being a "good" doctor.

Others focused on healing work with individual patients aligned with Gilligan's care orientation (although not related to gender here), but highlighting the problem of universal treatment in medical care:

"[Y]ou can't practice medicine for different socioeconomic groups. you practice medicine for the whole community" (Focus Group GP Participant).

To go beyond providing the best possible clinical care for each individual patient was thought to be dangerous, undermining the moral focus of doctoring. To start identifying and accounting for an individual patient's life within a social hierarchy was a slippery path. By caring for everyone equally, somehow blind to the wider social context at play in the illness trajectory of patients' lives, each individual patient will be assured of the care they need, and close attachment between doctor and patient will be maintained. Silence about social health inequities is actively at work here.

Silence, as intimated earlier, is maintained through collective endeavours of how individuals and professions respond (or do not) to issues [11]. Professions for example limit the scope of their member's attention, providing informal and formal rules of denial that shape notions of what is relevant to practice, providing the mechanism through which selected issues and situations remain nameless. Thus, professional associations do have a role to play in addressing moral matters, but their role is not our focus in this work. At the level of the individual encounter, Mishler has described the strategies through which silence can be managed by doctors:

"[P]hysicians controlled the flow of the clinical interview: (1) through their ways of asking questions; (2) by interrupting patients' efforts to say more than was asked for, often in the form of stories; and (3) by ignoring, that is, refusing to acknowledge or respond to, patients' accounts of the effects on their daily lives of symptoms of their illness"[5].

Such notions of socialised silence and denial shape encounters between individual family physician and patients.

There are two types of silence illustrated in the above examples. First there is a silence about the social basis of much of the illness and suffering encountered in primary care. Avoiding the reality that we are not all equal in the social lives we lead, or the health consequences of this, generates a sense of universalism whereby physicians pride themselves on delivering health care equally to everyone, but unintentionally ignore social health inequities. Second, there is a silence about the convincing evidence that doctors themselves are actively involved in generating and sustaining social hierarchies of illness care. This occurs through differential treatment of patients based on social and demographic characteristics such as race, ethnicity, gender and education [20-22].

In contrast to physicians, leaders of the profession and academics saw the matter of responding to social health inequities quite differently. While physicians tended to draw on a care orientation, this group constructed social health inequities as an issue of equitable and just access to medical care and the rights of vulnerable communities:

"[O]ur value system has been a bit lost. No-one is even talking about whose duty of care it is to provide care for disadvantaged sections of the community. Neither state nor medical schools seem to take it on" (External Interview Respondent).

Addressing health inequities for these participants meant mandating and regulating aspects of the work of individual practitioners as well as the profession as a whole. Physicians for example should monitor and be accountable for ensuring that preventive care reaches vulnerable and disadvantaged groups in their practice community. The physician's response is through providing access to care.

Here social health inequities is very much an issue within the remit not only of the profession but, importantly, individual doctors as well. Yet, the focus is more widely on the social structures and contexts that surround medical care and clinical encounters. The tension raised by this position rests is the potential to create an institutionalised and bureaucratised vision of medical care driven by monitoring and accounting that is at risk of undermining the very humanistic values that it seeks to defend. As May [23] describes, the rising levels of chronic care and the intrusion of funders and managers and systems for monitoring and accountability into the doctor-patient relationship are transforming the notion of "patient-hood" and the work of primary care physicians. Reponses to health inequities framed within a justice orientation tended to implicitly draw on such a regulated and highly specified vision of clinical care. The examples we have provided so far reveal that there are limits to both the justice and care orientations being used as a guide for action. This may be related to how respect for justice, beneficence, non-maleficence and autonomy can be interpreted by individuals holding these orientations as shown in Table 1.

**Table 1:** Expressions of the four principles of bioethics within the two moral orientations

	Social Justice and Human Rights	Care and compassion for the vulnerable
**Beneficence**	Doing good involves ensuring the individual gets the health care they need even when their social position limits their opportunities for health achievement (this may involve reorienting services: ensuring services are available, accessible and appropriate).	Doing good involves providing the best available clinical care to each individual within a compassionate, caring and empathic context.
**Non Maleficence**	Doing harm involves paying no attention to the social contextual factors at play in a patient's illness presentation and experience.	Doing harm involves changing the care one provides on the basis of a person's social position. Everyone should be treated in the same way.
**Autonomy**	Promoting autonomy involves helping individuals overcome the social limits that frame their choices through full information to promote access to clinical care.	Autonomy flows from the full attention, support and engagement of the clinician in a relational encounter.
**Justice**	Justice is premised on the notion of natural rights to equitable access to health care as an element of a free, dignified and meaningful life.	Justice will ensue if practitioners see beyond a patient's social context to the person within. Physicians have a duty to respond to inequities through caring for patients from all backgrounds.

Our re-examination of the data indicates that justice, beneficence, non-maleficence and autonomy [24] continue to underpin **both **the justice and care orientations. Although the care orientation is ethically preferable to the justice orientation in the way that the principles are articulated through connection, the problem of universality remains an issue. Justice would suggest that beneficence and non-maleficence are upheld by advocating for each patient, leveraging resources and access to care, premised on an understanding of the social and structural limits the patient lives within and the impact on illness. On the other hand, in the care orientation beneficence is upheld by ensuring the continuous healing presence of the physician, un-distracted by extraneous social and societal influences. Patients are free and autonomous only through the physician's pursuit of these apparently opposing strategies, which is a fairly one-sided view of the doctor-patient relationship. Justice will be done by righting inequities of access on the one hand, while through unconditional acceptance on the other.

Accounting for social context need not lead to a bureaucratic inhumane form of medical care or blindness to socioeconomic factors underpinning the health impact of social disadvantage. Experiential knowledge and biographical information, gathered over time allows a physician to treat the patient as a person, more than simply a means to an end [25]. Inequities and social disadvantage can be engaged with through sharing in patient's lives over time and helping those who are suffering and oppressed, not just knowing illness and treatment. However to achieve this, something more than the moral orientations and four bioethical principles is required. Unfortunately, normative ethical theories like virtue ethics, deontology and utilitarianism do not provide adequate guidance either.

## The internal morality of medical care: Pellegrino's ethical theory and Wilde's critique

To explain these limitations of normative ethical theories, Pellegrino for example has long claimed that the physician-patient encounter is "the starting point for a philosophy of medicine and the root of its internal morality" [26]. Medical care has teleological ends that refer to the good of the patient and their return from poor health to better health; ideally the "making whole again" of the sick. Where such a transformation is not possible, caring and comforting, simply being a part of how patients live with illness or disease is medicine's healing purpose. Pellegrino argues that this internal morality springs from the essence of medical care, its nature; "to care and heal". He sees the common meaning of encounters between healer and patient across cultures, place and history as evidence of the intrinsic phenomenological nature of clinical medicine as a form of practice with its own internal morality. Pellegrino asserts that to argue otherwise is to relegate medical practice to being "an instrument of social and political purpose and the physician [to the role of] social functionary" [[26]: 177]. Pellegrino's view is that the virtuous physician hears the call for help from the patient and responds.

Wildes [27], in a critique of Pellegrino's philosophy, highlights the potential problem of 'silence' contained within this position however. Wildes argues that "to focus solely on the physician-patient encounter is an incomplete phenomenology (of medical practice) since the encounter is shaped by the presence of others, as well as the structures that make the encounter possible" [[27]:79]. For Wildes and others medical care is at its core a form of social practice, intimately entwined with the social structures, relations of power and institutional settings that make it possible and without which it would not exist. In this view any ethical theory or philosophy of medical care must engage with the social and societal nature of medical practice or risk irrelevance to both practitioners and patients.

Pellegrino [26] rightly worries about the outcome of allowing a socially constructed purpose for medical care defined external to the profession and its calling. For Pellegrino the role of the physician is to heal the individual patient. Responsibility for addressing the health effects of societal inequities lies outside of medicine, outside of its moral remit. Yet the elephant remains in the room and the question persists -- how should physicians respond to individual patients suffering the health effects of social inequity? If the purpose of medicine is a duty to make whole again, there must be an engagement with the social dimensions of patient's lives causing them ill-health.

The problem of how to respond to social health inequities is not resolved by turning to utilitarianism either. The theoretical focus to act in the interests of the greater good for the greatest number means that if the patient's health with its basis in social inequity is not considered a part of the greater good, there is no great compulsion on the physician's part to act. Adopting a deontological position that it is one's duty to act does not bring the elephant out of the room either. Applying the categorical imperative of "acting only according to the maxim that you can will to become universal law" [28] cannot solve the highly individualised nature of inequities.

Even the modern day version of the Hippocratic Oath, the Geneva Declaration expresses a position of equal, universal treatment and the physician's obligation to care:

"[T]he health of my patient will be my first consideration [...] I will not permit considerations of religion, nationality, race, party politics or social standing to intervene between my duty and my patient" [29].

This notion of universal and equal treatment of patients regardless of their social position suggests physicians should remain impartial and "practice medicine for the whole community not socio-economic groups". However we have learned from Aristotle's position on justice that universal treatment represents one of the greatest injustices - the equal treatment of unequals does not ensure distributive justice [30]. If we treat everyone equally then social inequities remain effectively denied and silenced.

It is surprising given the quotidian nature of this issue that the primary care profession has not engaged in vigorous debate about or reached a consensus on the extent of its responsibility in relation to social disadvantage. Our critique indicates that we still need to develop ethical guidance on this matter. What may be required is a conceptual shift in how social health inequity is understood within the medical encounter.

## Beyond moral orientations

For family physicians there are few ethical theories that can guide action to transform and address social inequities. Appreciating the moral orientations and the nuances of the way they are at work helps to illuminate where physicians place their boundaries of moral responsibility in relation to social health inequities, but it does not ensure active engagement. As a set of reductionist propositions they cannot "articulate well what, in the end, influences most what we believe and how we are in the world" [[19]:207]. Rather than the application of sets of principles that ultimately end up outweighing each other [31], an important starting point is the grounded development of an ethical theory applicable to the day-to-day clinical encounter.

Patient-centred practice [32] might be seen as offering potential for resolution of some of the difficulties we have outlined. Here care is tailored according to a patient's context, accounting for the whole person needs. There is an emphasis on shared decision-making, a holistic understanding of the patient and a complete bio-psycho-social assessment of a patient's problem. This approach attempts to deal with the inequities of power between patients and physicians and offers a framework for bringing the patient's social world into the everyday encounter. However, in reality physicians tend to deal with the bio-psychological and less with the social [33] and the patient-centred model remains excessively individualised. The challenge lies in delineating the boundaries around how and how far physicians can go in addressing underlying social factors at play in a patient's ill-health. Some of this is related to how our relationships with others are understood.

## Forms of togetherness: developing a response

The three forms of togetherness Bauman [13] described go some way to developing a response to this dilemma of social health inequities. This is because the three kinds of encounters *being-aside*, *being-with *and *being-for *can assist to explain and understand how we engage with others in ways that yield different moral consequences. Understanding this engagement and the moral consequences of this can be used to break the silence on social health inequities. In this final section we draw on the example of uninsured patients in the U.S. to illustrate the forms of togetherness and their application to this one issue of the effects of social health inequities.

In a crowd at a railway station, sitting in adjacent cars in a traffic jam, passing familiar but unknown faces, to and fro, each day is *being-aside*. *Being-aside *is an "on the side" encounter, where other people are recognised simply as co-present entities with no vested interests. While we might naturally feel that *being-aside *is antithetical to medical care and practice and rarely seen in that setting, when it comes to responding to social health inequities many do remain on the side. The case of uninsured patients in the U.S. in need of urgent health care but unable to gain access resembles the encounter of *being-aside*.

The movement into *being-with *at least signals the beginning of recognition of others. This relation is exemplified for instance when a physician treats an uninsured patient because of their call for help, and because it is the right thing to do. In this response, there is a beginning recognition that the other needs assistance. Bauman says the entities which were on the side gain attention and take shape as persons, however:

"*[b]eing-with *is still a mis-meeting of incomplete beings, of deficient selves [where] not more of the self tends to be deployed in the encounter than the topic-at-hand demands; and no more of the other is highlighted than the topic at hand permits" [[13]:50].

*Being-with *reflects a relationship based on transaction and utility, an exchange of goods, a form of quid-pro-quo, and user pay systems. Recognition of the other is based on a cognitive expression of need; responsibility ends at the completion of the transaction. In the case of uninsured patients, *being-with *means the patient continues to have no access to full health care services. There has been a temporary alleviation of need and while this encounter has been encouraged by relational qualities like compassion and sympathy, the more enduring feature of a sustained responsibility is not present.

Certainly, the moral consequences of *being-with *are starkly different from *being-aside*. Indeed, it is not difficult to see here some of the moral consequences of fragmented, "de-humanised" health care systems. Patients and doctors face pressures to be efficient in their dealings. Modern patients are expected to be active, resourceful and prudent in their use of health care resources [34]. Doctors must meet quality and administrative targets, and are increasingly accountable to funders for their work. How is it that we move beyond *being-with *to the ideal relation of *being-for?*

*Being-for *is premised on absolute recognition of our otherness (alterity) and a dialogical relation of interconnection shaped by the inability to finalise another by speaking for them or trying to become the other. *Being-for *is an ontological space of togetherness, an ideal form of togetherness, that occurs in the act of transcendence, from seeing and *being*-*with *"something" to *being-for *"someone". Physicians who treat uninsured patients might share some of these qualities, but *being-for *is different from having empathy for another's suffering where "my projecting what would make me feel better onto you, or my fusing with suffering could result in unification" [[35]:116]. In *being-for *the recognition of the human face of the other is an embodied feeling which compels one to respond to another's needs, but not to the point of having all of the answers and closing the relationship off. *Being-for *makes dialogue between two people possible and there is a realisation that "who you are depends on who I am" [[35]:118].

*Being-for *recognises the preciousness of the other, their full properties and their identity and it is a relation that does mean social health inequities can be an actively acknowledged as part of the relationship and our alterity. For example, Forester and Heck [36] complete a simple act of *being-for *by talking with uninsured patients about the differences in the costs of accessing primary care services compared to their health deteriorating to the point of needing hospitalisation. Rather than leaving this unsaid as a matter of their patient's choice, the physicians introduce dialogue about this because they recognise that who they are affects who the patient is and what they will become. They also recognise that caring for uninsured patients is part of a higher moral purpose, for them it is a reminder of what being a family physician is all about.

While it may be said that recognising our differences still does not address the issue of responding directly to inequities, a change such as that outlined by Forester and Heck in how we respond to the issue of social health inequities has moral consequences. This ontological shift in fact makes inequities and differences a part of our relationships, and although the example we have provided relates to the particular case of the insured in the U.S, there are patients who have financial difficulties in accessing various parts of the health service in all health care systems. There are responses that physicians can make to their needs which begin with the acknowledgement that we do not stand together universally, devoid of socio-economic grouping and cultural context. To make even this small shift there are barriers to overcome. For example professional training of doctors fosters distance and encourages neutrality and silence on such matters [37], political systems encourage choice and individual autonomy. Developing the relation of *being-for *is possible however within the day to day primary care medical encounter.

This need not be overwhelming for clinicians, faced with a full waiting room of patients and the responsibility and seeming impossibility of acting on the social and societal forces embodied in each. "Being for" starts before and goes beyond any of these. *Being-for *does not ignore the critical exchanges that must occur as day to day illness care proceeds, rather it provides a way of being together that infuses these. For the physician it demands a reflexive awareness of the way they may contribute to shaping the encounter, sensitivity to questions that might bring foreclosure, limit responses and create silence. It also demands that patients too come to recognise the physician with their uniqueness and alterity.

"Being with" in a transactional clinical exchange may improve access to aspects of care and bring compliance, adherence and achievement of quality care targets, but crossing over to *being-for *may provide a basis for action on social health inequities, bring personal reward and work toward a better health care system.

We have outlined that the impartiality of principled based ethics does not allow us to see or share the embodied nature of social injustice and suffering in patients and physician in their togetherness. Institutions fall short in their responses because they either see social disadvantage as the individual's responsibility or it is not within the remit of the greater good. Before we can adequately grapple with this question, however, it seems necessary to re-acknowledge the moral imperative physicians and their professional associations have to respond to social health inequities in their everyday practice and the possibility of doing so. How we think about this problem of social health inequities needs much greater examination. The application and implementation of these three forms of togetherness can highlight some of the moral consequences of the different kinds of relationships doctors and patients engage in.

## Competing interests

The authors declare that they have no competing interests.

## Authors' contributions

JF was lead investigator on the study on which this paper draws. This paper was developed through successive drafts, iterations and meetings between both JF and VP, who contributed equally to the final manuscript.

## References

[B1] Wilkinson R Marmot M Social Determinants of Health: The Solid Facts Social Determinants of Health: The Solid Facts 2003 2 WHO

[B2] Gunn JM Naccarella L Palmer VJ Kokanovic R Pope C Lathlean What is the Place of Generalism in the 2020 Primary Care Team? What is the Place of Generalism in the 2020 Primary Care Team? 2007 Australian Primary Health Care Research Institute

[B3] Palmer VJ Naccarella L Gunn JM Are you my specialist or generalist of my care? New Zealand Family Physician 2007 34 394 397

[B4] Stange KC The Problem of Fragmentation and the Need for Integrative Solutions Ann Fam Med 2009 7 100 103 10.1370/afm.971 19273863 PMC2653966

[B5] Mishler EG Patient stories, narratives of resistance and the ethics of humane care: a la recherche du temps perdu Health 2005 9 431 451 10.1177/1363459305056412 16144787

[B6] Pellegrino E Ethics and the Moral Center of the Medical Enterprise Bulletin of the New York Academy of Medicine 1978 54 625 640 276400 PMC1808197

[B7] Bower P Gask L May C Mead N Domains of consultation research in primary care Patient Education & Counseling 2001 45 3 11 10.1016/S0738-3991(01)00117-3 11602363

[B8] Tuckett D Boulton M Olson C Williams A Meetings between experts: An approach to sharing ideas in medical consultations 1985 London: Tavistock

[B9] Skirbekk H Negotiated or taken-for-granted trust? Explicit and implicit interpretations of trust in a medical setting Medicine Health Care and Philosophy 2009 12 3 7 10.1007/s11019-008-9142-2 18512132

[B10] Cegala D The Impact of patients' participation on physician's patient-centered communication Patient Educ Couns 2009 77 202 208 10.1016/j.pec.2009.03.025 19395225

[B11] Zerubavel E The elephant in the room: silence and denial in everyday life 2006 New York: Oxford University Press

[B12] Harris M Knowlden S Clinical perspective: a general practitioner response to health differentials 1999 Sydney: Australian Centre for Health Promotion

[B13] Bauman Z Life in Fragments: Essays in Postmodern Morality 1995 Oxford, Cambridge: Blackwell

[B14] Levinas E Totality and Infinity: An Essay on Exteriority 1969 Pittsburgh: Duquesne University Press

[B15] Furler J Harris E Harris M Naccarella L Young D Snowdon T Health inequalities, physician citizens and professional medical associations: an Australian case study BMC Medicine 2007 5 23 10.1186/1741-7015-5-23 17697318 PMC1995197

[B16] Gilligan C In a different voice: psychological theory and women's development 1993 Cambridge, Mass.: Harvard University Press

[B17] Friedman M What are friends for?: feminist perspectives on personal relationships and moral theory 1993 Ithaca, NY: Cornell University Press

[B18] Self DJ Jecker NS Baldwin DC Jr The moral orientations of justice and care among young physicians Cambridge Quarterly of Healthcare Ethics 2003 12 54 60 10.1017/S0963180103121068 12625201

[B19] Little MO Care: From Theory to Orientation and Back J Med Philos 1998 23 190 209 10.1076/jmep.23.2.190.8922 9638569

[B20] van Ryn M Burke J The effect of patient race and socio-economic status on physicians' perceptions of patients Soc Sci Med 2000 50 813 828 10.1016/S0277-9536(99)00338-X 10695979

[B21] van Ryn M Fu SS Paved with good intentions: do public health and human service providers contribute to racial/ethnic disparities in health? American Journal of Public Health 2003 93 248 255 10.2105/AJPH.93.2.248 12554578 PMC1447725

[B22] Lutfey KE Campbell SM Renfrew MR Marceau LD Roland M McKinlay JB How are patient characteristics relevant for physicians' clinical decision making in diabetes? An analysis of qualitative results from a cross-national factorial experiment Soc Sci Med 2008 67 1391 1399 10.1016/j.socscimed.2008.07.005 18703267 PMC2582199

[B23] May C Chronic illness and intractability: professional-patient interactions in primary care Chronic Illness 2005 1 15 20 17136927 10.1177/17423953050010011201

[B24] Beauchamp TL Childress J Principles of Biomedical Ethics 1994 New York: Oxford University Press

[B25] Baik SY Bowers BJ Oakley LD Susman JL What comprises clinical experience in recognizing depression?: The primary care clinician's perspective Journal of the American Board of Family Medicine 2008 21 200 210 10.3122/jabfm.2008.03.070258 18467531

[B26] Pellegrino ED Philosophy of medicine: Should it be teleologically or socially construed? Kennedy Institute of Ethics Journal 2001 11 169 180 10.1353/ken.2001.0015 11708333

[B27] Wildes KW The crisis of medicine: Philosophy and the social construction of medicine Kennedy Institute of Ethics Journal 2001 11 71 86 10.1353/ken.2001.0008 12166446

[B28] Preston N Understanding Ethics 1996 1 Sydney: The Federation Press

[B29] Declaration of Geneva Adopted by the General Assembly of World Medical Association at Geneva Switzerland 1948

[B30] Aristotle (Translated Ross) WD RM H Nicomachean Ethics. Book V, Chapters 2-4 Great Books of the Western World: Aristotle II 1952 Chicago: The University of Chicago

[B31] Guillemin M Gillam L Telling moments: everyday ethics in health care 2006 East Hawthorn, Vic.: IP Communications

[B32] Stewart M Brown JB Weston WW McWhinney IR McWilliam CL Freeman TR (Eds) Patient-centered medicine: transforming the clinical method 2003 Oxford: Radcliffe Medical Press

[B33] Dowrick C May C Richardson M Bundred P The biopsychosocial model of general practice: rhetoric or reality? Br J Gen Pract 1996 46 105 107 8855018 PMC1239540

[B34] May C Self-manangement of chronic conditions: Re-engineering patient-hood Chronic Illness 2006 2 15 16 10.1177/17423953060020010601 17175676

[B35] Frank AW The Renewal of Generosity: Illness, Medicine and How to Live 2004 Chicago: The University of Chicago Press

[B36] Forester RA Heck RJ What you can do to help your uninsured patients Fam Pract Manag 2009 16 21 24 19751039

[B37] Candib N Medicine and the family: a feminist perspective 1995 New York: Basic Books

